# MDG-1, a Potential Regulator of PPARα and PPARγ, Ameliorates Dyslipidemia in Mice

**DOI:** 10.3390/ijms18091930

**Published:** 2017-09-08

**Authors:** Xu Wang, Linlin Shi, Sun Joyce, Yuan Wang, Yi Feng

**Affiliations:** 1 Engineering Research Center of Modern Preparation Technology of TCM, Shanghai University of Traditional Chinese Medicine, Shanghai 201203, China; xuwang0415@126.com (X.W.); shilinmochen@126.com (L.S.); 2 Department of Biological Sciences, National University of Singapore, Singapore 117543, Singapore; e0006838@u.nus.edu

**Keywords:** MDG-1, hyperlipidemia, gene microarray, PPAR

## Abstract

Hyperlipidemia is a serious epidemic disease caused by lipid metabolism disorder, which is harmful to human health. MDG-1, a β-d-fructan polysaccharide extracted from *Ophiopogon japonicus*, has been shown to improve abnormal blood lipid levels and alleviate diabetes. However, the underlying mechanism on hyperlipidemia is largely unknown. In this study, male C57BL/6 mice were randomly separated into three groups, respectively: low-fat diet (Con), high-fat diet (HFD), and high-fat diet plus 5‰ MDG-1 (HFD + MDG-1). Body weight was measured and the serum lipid levels were analyzed. Using gene microarray, various core pathways, together with levels of gene expression within hepatocytes, were analyzed. RT-PCR was used to confirm the identity of the differentially expressed genes. MDG-1 could prevent obesity in HFD-induced mice and improve abnormal serum lipids. Besides, MDG-1 could regulate hyperlipidemia symptoms, specifically, and decrease fasting blood glucose, improve glucose tolerance, and ameliorate insulin resistance. According to results from gene microarray, most of the identified pathways were involved in the digestion and absorption of fat, biosynthesis, and catabolism of fatty acids as well as the secretion and biological synthesis of bile acids. Furthermore, MDG-1 may act upon peroxisome proliferator-activated receptors (PPAR) α and γ, activating PPARα whilst inhibiting PPARγ, thus having a potent hypolipidemic effect.

## 1. Introduction

Hyperlipidemia is a group of disorders characterized by an excess of lipids in the bloodstream. Apart from genetic factors like familial hypercholesterolemia [1,2], hyperlipidemia is closely associated with less healthy living habits and dietary preferences. Mounting evidence shows that hyperlipidemia increases the risk of chronic metabolic disorders, such as obesity, cardiovascular disease, and particularly type II diabetes, resulting in high mortality rates [3,4,5,6]. Moreover, hyperlipidemia is a widely-accepted risk factor for elevated blood glucose levels and insulin resistance [7,8,9], implying more attention should be given to the regulation of lipid metabolism in diabetic patients. As an epidemic public problem, research on lipid-control therapies must be accelerated.

Lipid metabolism has emerged as an important modulator of hyperlipidemia and abnormal fat. The lipid-activated transcription factors PPAR are members of the nuclear receptor superfamily that have a well-defined role in regulating lipid homeostasis and metabolic diseases [10]. Peroxisome proliferator-activated receptors (PPARs) comprised of three different isoforms, namely, PPARα, PPARβ, and PPARγ, play a vital role in the biological processes of the metabolic syndrome, including fat generation, lipid balance regulation, energy metabolism, insulin sensitivity, cell differentiation, and immune response [11,12,13,14]. Accumulating studies demonstrate that these nuclear receptors (PPARα, PPARβ, and PPARγ), all have well-documented roles in lipid and glucose metabolism. Current reports suggest that okra polysaccharides (OP) have therapeutic effects on metabolic diseases via the inhibition of LXR and PPAR signaling [15], and *Astragalus* polysaccharides could regulate gene expression of PPARα and its target genes to improve lipid metabolism [16]. Besides, polysaccharides from *Rosae Laevigatae Fructus* could improve hyperlipidemia possibly through regulating PPAR-mediad lipid metabolism [17]. These suggest that polysaccharides modulate hyperlipidemia through the regulation of PPAR signaling.

Based on our previous studies, MDG-1 [18], a β-d-fructan polysaccharide with an average molecular weight of 3400 Da, extracted from the root of *Ophiopogon japonicus* (Radix *Ophiopogonis japonici*), a traditional Chinese medicine, has a beneficial effect on blood glucose levels and body weight in diet-induced obese mice (DIO) [19,20] or the model of type 2 diabetes *ob*/*ob* mice [21]. MDG-1 could also improve serum lipid levels and regulate the synthesis, secretion, and reabsorption of bile acids [19]. Thus, in our research, we aimed to test that MDG-1 may improve high-fat diet (HFD)-induced obesity, dyslipidemia, and glucose resistance. Besides, in consideration of the gene chip with high throughput that could determine the different functional status and core gene expressions at the same time, we used the Affymetrix Mouse Gene 2.1 ST Array Strip (Thermo Fisher Scientific, Waltham, MA, USA) gene chip to screen significantly different genes and discovered gene expression profiles between the HFD group and the MDG-1 prevention group, thus exploring more deeply the mechanism of MDG-1 on hyperlipidemia.

## 2. Results

### 2.1. MDG-1 Blocks Obesity in DIO Mice

**Figure 1:**
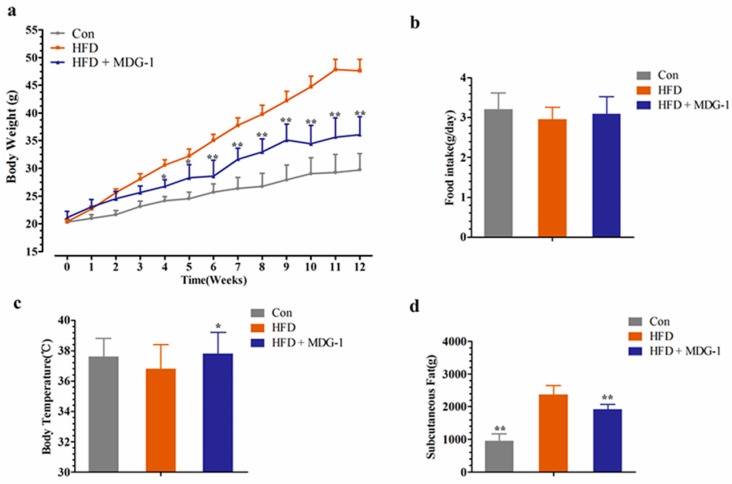
MDG-1 blocks obesity in diet-induced obese (DIO) mice. (**a**) Body weight gain; (**b**) food intake amount; (**c**) body temperature; (**d**) mass of subcutaneous fat. Data were presented as means ± SD (*n* = 8). * *p* < 0.05, ** *p* < 0.01 vs. high-fat diet (HFD) group.

Compared to the control (Con), the body weight of the HFD group increased dramatically (Figure 1a). As illustrated in Figure 1a, in comparison with the HFD group, MDG-1 supplemented with a high-fat diet (HFD + MDG-1) obviously lessened body weight after three weeks prevention (this trend was observed throughout the whole experiment). The food intake of the MDG-1 group was consistent with that of the HFD group (Figure 1b), implying that the reduction in weight was due to the effects of MDG-1 and not decreased energy intake. Interestingly, MDG-1-treated mice showed a significantly higher body temperature compared to the DIO mice (Figure 1c), indicating that MDG-1 may enhance energy expenditure.

**Figure 2:**
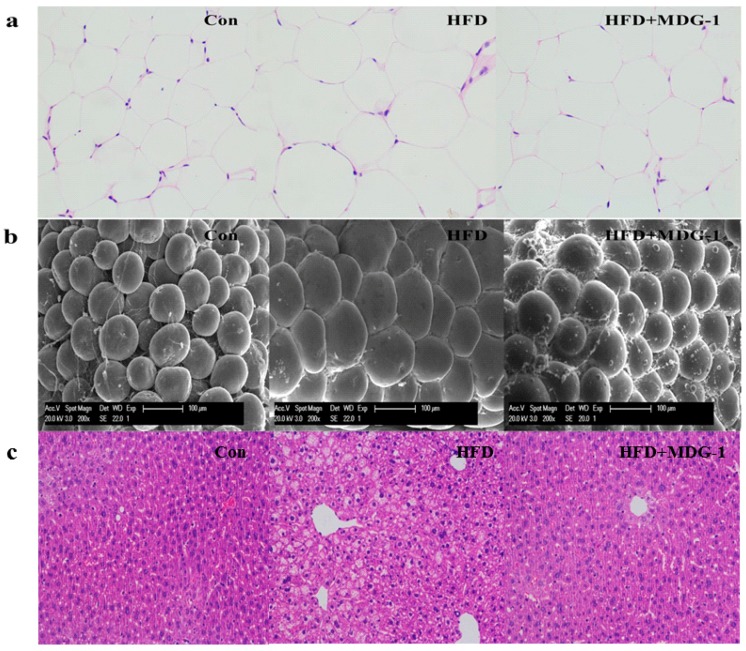
Images of white adipocytes and liver tissue. (**a**) Hematoxylin and eosin (H&E) staining of adipose tissue (×200); (**b**) electronic scanning microscopy of adipose tissue (×200); (**c**) H&E staining of liver tissue (×200).

To further confirm that the intervention of MDG-1 could prevent fat accumulation of obese mice (the weight and size of subcutaneous fat was measured and analyzed). As expected, the mice of the MDG-1 group apparently decreased subcutaneous fat weight compared to that of the HFD group (Figure 1d). In addition, the Hematoxylin and Eosin (H&E) staining (Figure 2a) and Electronic Scanning Microscopy Assays (Figure 2b) showed that the size of the adipocytes of the MDG-1-treated mice was reduced. These results implied that MDG-1 could protect against high fat, diet-induced fat accumulation. Besides, liver histology was detected by H&E staining (Figure 2c). In the H&E sections, the mice fed the HF diet developed obvious steatosis and vacuolization compared to the Con group, whereas MDG-1 treatment notably ameliorated macrovesicular steatosis of the liver compared to the HFD group. Taken together, these results indicated that MDG-1 could increase energy expenditure and reduce fat accumulation and hepatic steatosis, thus regulating body weight in the high-fat diet mice.

### 2.2. MDG-1 Attenuates Dyslipidaemia in DIO Mice

**Figure 3:**
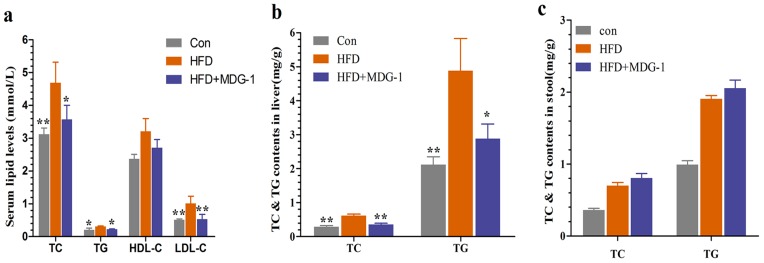
MDG-1 attenuates dyslipidaemia in DIO mice. (**a**) Serum total cholesterol (TC), triglyceride (TG), high-density lipoprotein cholesterol (HDL-c), low-density lipoprotein cholesterol (LDL-c); (**b**) TC and TG contents in the mice liver; (**c**) TC and TG contents in the mice stool. Data were presented as means ± SD (*n* = 8). * *p* < 0.05, ** *p* < 0.01 vs. the HFD group.

To further verify that MDG-1 could moderate many of the symptoms present in metabolic syndromes, serum lipid levels were assayed. Figure 3a showed that MDG-1 displayed markedly lower levels of total cholesterol (TC), total triglyceride (TG), and low density lipoprotein cholesterol (LDL-c) than those of the HFD group, whereas the high density lipoprotein cholesterol (HDL-c) level remained unchanged. Meanwhile, we determined TC and TG contents in the stool and liver. The fecal TC and TG levels of the MDG-1 group were increased to a certain extent compared to the HFD group. Moreover, TG and TC levels in the liver of the MDG-1 group were markedly ameliorated as compared to those in the HFD group, implying that MDG-1 could notably lower lipid accumulation in the liver tissue (Figure 3b,c). These results showed that MDG-1 prevention had a positive effect on the regulation of lipid metabolism.

### 2.3. MDG-1 Improves Glucose Tolerance and Insulin Resistance in Obese Mice

**Figure 4:**
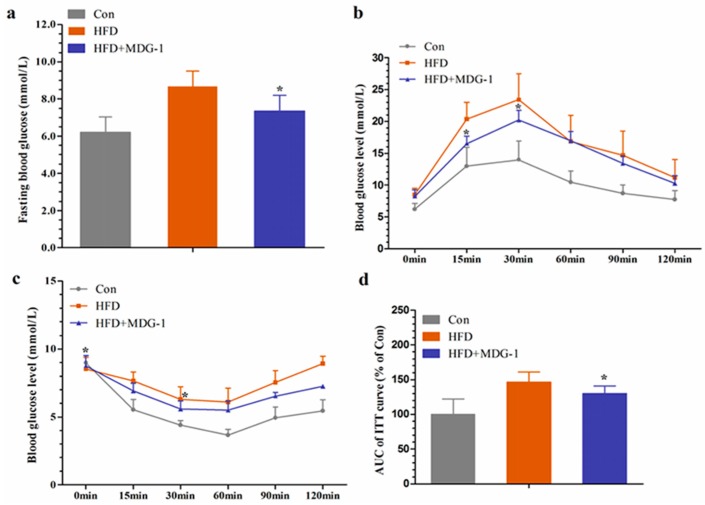
MDG-1 improves glucose tolerance and insulin resistance in obese mice. (**a**) Fasting blood glucose level; (**b**) glucose tolerance test (GTT); (**c**) insulin tolerance test (ITT); (**d**) quantification of the area under the ITT curve. Data were presented as means ± SD (*n* = 8). * *p* < 0.05, ** *p* < 0.01 vs. HFD group.

Hyperlipidemia is closely related to diabetes. Considering that MDG-1 could adjust the disturbances of lipid metabolism, we investigated whether MDG-1 could improve hyperglycemia in HF mice. We determined fasting glucose at the end of the experiment. The blood glucose level of the HFD group was considerably higher than that of Con group, whereas the blood glucose of the HFD + MGD-1 group was depressed compared to HFD group. Meanwhile, the glucose tolerance test showed that MDG-1 markedly lowered the blood glucose levels at 15 min and 30 min in HF-induced mice (Figure 4b), which suggested that MDG-1 notably improved glucose tolerance.

To investigate whether long-term MDG-1 intervention could improve insulin resistance in obese mice, an insulin tolerance test was conducted. With 0.75 U/kg insulin, we measured blood glucose levels at 0, 15, 30, 60, 90, and 120 min intervals (Figure 4c). Experimental results showed that compared with the HFD group, the blood glucose level of mice was significantly lowered with the MDG-1 supplement at 0 min and 30 min, and the blood glucose area under the curve of the MDG-1 group decreased by 39.6% at 120 min (Figure 4d). The results showed that MDG-1 could increase the sensitivity of insulin and ameliorate insulin resistance caused by hyperlipidemia.

### 2.4. MDG-1 Effects the Expression of Lipid Genes in HFD-Fed Mice by Gene Chip Technology

**Figure 5:**
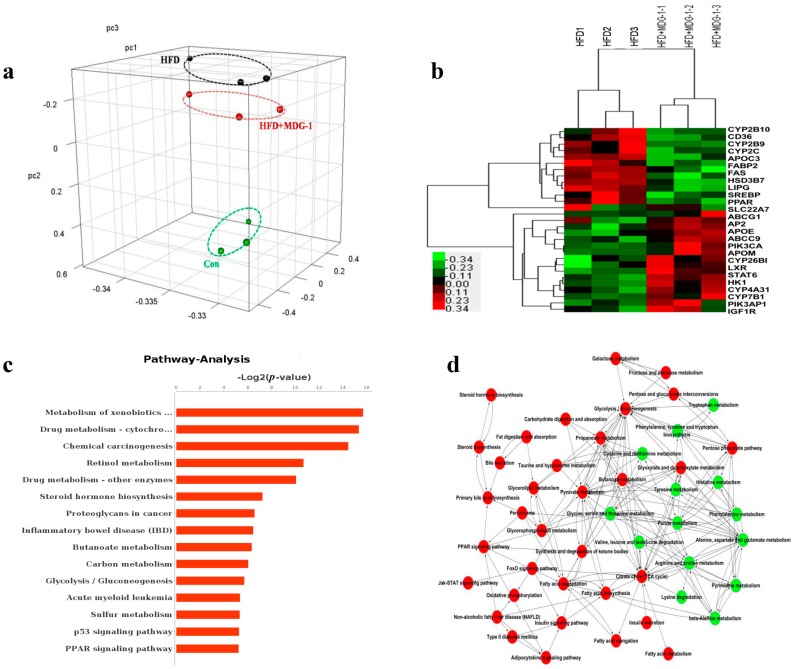
MDG-1 effects the expression of lipid genes in HFD-fed mice by gene chip technology. (**a**) 3D-PCA; (**b**) the different genes clustering diagram; (**c**) pathway analysis; (**d**) the core pathway network. Data were presented as means ± SD (*n* = 3). * *p* < 0.05, ** *p* < 0.01 vs. the HFD group.

To explore the underlying mechanism behind MDG-1’s intervention in lipid metabolism disorder, we analyzed the gene microarray of the livers within the HFD group and the MDG-1 group using the Affymetrix Mouse Gene 2.1 ST Array Strip (Thermo Fisher Scientific, Waltham, MA, USA). This ultimately helped to explicate the mechanism of MDG-1 at the genetic and molecular level. We found that after 12 weeks of prevention trials, the MDG-1 intervention group was clustered together, and was relatively distant from the HFD group but close to the normal group based on the results of 3D-PCA, suggesting a trend towards recovery when using MDG-1 against HFD-induced obesity in experimental mice (Figure 5a).

Based on the analysis of 3D-PCA, we obtained the different genes clustering diagram (Figure 5b) to depict the variation of genes between liver samples taken from the MDG-1 group and the HFD group. Figure 5b revealed that the 27 genes found in the samples were expressed differentially in the MDG-1 group compared to the HFD group. Among them, 15 genes were significantly up-regulated and 11 genes were significantly down-regulated through the intervention of MDG-1. Interestingly, MDG-1 played a major role in regulating the expression of a myriad of genes affiliating the PPARs family. Specifically, MDG-1 increased the expression of LXRα, LXRβ, CYP7B1, ApoE, and SREBP-1c and inhibited the expression of PPAR, FAS, ACC, CD36, and AP2.

Furthermore, pathway analysis was performed on the basis of differential expression genes to obtain targeting signal pathways which the genes participated in. Figure 5c exhibited that differentiated signaling pathways mainly contained short-chain fatty acids (SCFAs) metabolism, steroid hormone biosynthesis, and the peroxisome proliferator activated receptor (PPAR) signaling pathway. Next, according to the selected pathways, the pathway-act-network was built via gene microarray to discover core pathways and the regulation of various signaling (Figure 5d). Most of the core pathways were involved in the digestion and absorption of fat, biosynthesis, the catabolism of fatty acid, the secretion and biological synthesis of bile acids, the PPAR signal pathway, amino acid pathways, and short-chain fatty acids metabolism, all of which were closely correlated with lipid metabolism. We inferred that MDG-1 supplementation in HF mice reversed the symptoms of dislipidemia through the alteration of interactions between multiple pathways, as described above.

### 2.5. MDG-1 Regulates Gene Expression of PPARs, LXR, and the Target Genes

**Figure 6:**
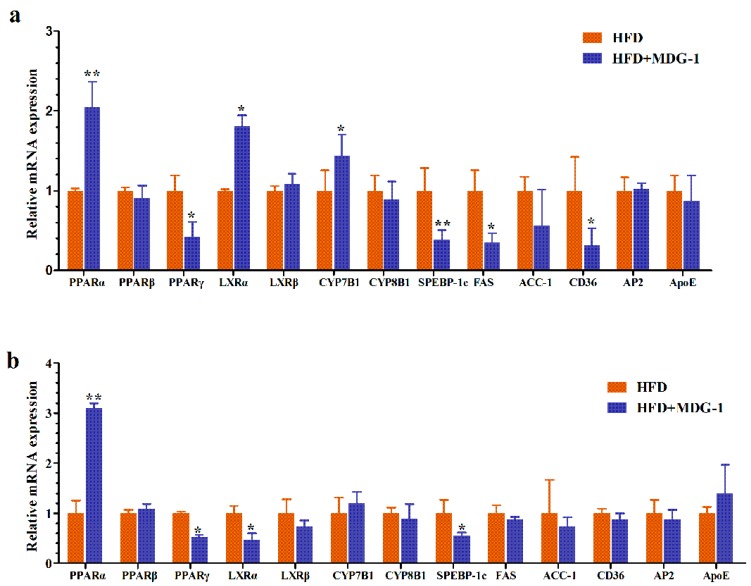
MDG-1 regulates gene expression of peroxisome proliferator-activated receptors (PPARs) and the target genes. (**a**) The mRNA expression levels of the genes in liver; (**b**) the mRNA expression levels of the genes in white fat. Data were presented as means ± SD (*n* = 8). * *p* < 0.05, ** *p* < 0.01 vs. the HFD group.

Based on results of gene microarray, quantitative real-time PCR (qPCR) analysis was used to confirm the differentially expressed genes (*PPARα*, *PPARβ*, *PPARγ*, *LXRα*, *LXRβ*, *CYP7B1*, *CYP8B1*, *ApoE*, *SREBP-1c*, *FAS*, *ACC*, *CD36*, and *AP2*) in vitro. Compared with the HFD group, we found that the mRNA expression levels of *PPARα* were significantly increased, whereas the gene expression levels of *PPARγ* were suppressed observably with MDG-1 intervention (Figure 6a), implying that MDG-1 could improve hyperlipidemia through regulating PPARs signaling. This was largely within our expectations. Besides, MDG-1 could regulate the target genes of *PPARα* and *PPARγ*. Figure 6a showed that within the liver, the mRNA expression levels of *LXRα*, *CYP7A1*, and CD36 were significantly increased, while *FAS* and *SREBP-1c* mRNA expression levels were noticeably suppressed in HFD + MDG-1-treated mice. Moreover, we measured the mRNA levels of *PPARα*, *PPARγ*, and their target genes in adipose tissues (Figure 6b). Consistently, we found that MDG-1 could enhance the mRNA levels of *PPARα* and *LXRα*, whist reducing the expression of *PPARγ* and *SREBP-1c* in white fat. Our data suggested that MDG-1 was a *PPARα* agonist and a *PPARγ* antagonist; it could moderate the PPARs pathway, therefore ameliorating hyperlipidemia in HFD-fed mice.

## 3. Discussion

The recent decades demonstrated excessive caloric intake and sedentary living habits, leading to increasing energy imbalances and ultimately resulting in obesity as well as hyperlipidemia (as public health problems, obesity, and hyperlipidemia are the main incentives of metabolic disorders (MetS) such as diabetes and hypertension with relatively high mortality [6,22,23]). Here, our test shows that MDG-1 has a protective effect on abdominal obesity and the disturbance of lipid metabolism. Moreover, based on hepatic gene microarray and quantitative real-time PCR (qPCR), we could reveal the mechanism of MDG-1 on the prevention of hyperlipidemia in HF diet-fed mice.

In the study, MDG-1 could markedly block the body weight gain in HF diet-induced C57BL/6 mice even when the food intake was not suppressed. This suggested that the body weight reduction by MDG-1 was not due to the lower caloric intake. It is also observed that the body temperature was significantly increased in the MDG-1-treated group as compared to the HFD-group; this result is consistent with our previous findings [24]. Furthermore, MDG-1 treatment resulted in a significant decrease in fat weight as well as adipocyte size. Excessive growth of adipose tissue results in obesity, which includes hyperplastic and hypertrophic [25]. MDG-1 could improve serum lipid levels and markedly lower lipid accumulation and steatosis in the liver tissue in HF diet-fed mice. Hyperlipidemia is a major pathogenic factor for diabetes, because obesity and high lipid levels stimulate excess glucose accumulation. Other than the above-mentioned effects, MDG-1 is also effective at lowering fasting blood glucose and improving glucose tolerance, as well as at ameliorating insulin resistance in HF diet-induced mice. Hence, MDG-1 could be a helpful option to prevent hyperlipidemia.

According to the results of gene microarray and pathway analysis, MDG-1 could regulate some signaling pathways which are closely linked to obesity and lipid metabolism including the digestion and absorption of fat, the biosynthesis and catabolism of fatty acid, and the secretion and biological synthesis of bile acids. Besides, MDG-1 intervention could alter the metabolism of SCFAs and amino acids. In our previous study, MDG-1 treatment could up-regulate the levels of SCFAs (especially butanedioic acid) and slightly alter the contents of amino acids based on the GC-TOF/MS analysis of fecal samples [24]. Moreover, researches indicated that SCFAs [26,27] and some amino acids [28,29] have been proven to impact de novo synthesis of lipids and glucose. Taken together, the core signaling pathways which MDG-1 influenced might interact together, thus exerting beneficial effects on obesity and abnormal lipids in HF mice.

More significantly, based on results from the gene chip and qPCR, we found MDG-1 had a large regulatory effect on PPARs expression. Specifically, MDG-1 intervention was found to activate the expression of *PPARα*, inhibit the expression of *PPARγ*, and regulate the expression levels of their target genes. *PPARα* mainly influences fatty acid metabolism and its activation lowers lipid levels, whereas *PPARγ* is mostly involved in the regulation of the adipogenesis, energy balance, and lipid biosynthesis [11]. Accumulating evidence has confirmed that promoting the expression of *PPARα* and its target genes is one of the mechanisms to improve glucose and lipid metabolism disorders in mice induced by high fat meals [30,31]. *Cyp4a10* and *Cyp4a14* are target genes for *PPARα*, which promote the oxidation of fatty acids [32]. In our study, we found that the expression levels of *PPARα* and *Cyp4a10*, *Cyp4a14*, and *aP2* in the liver tissue of HFD + MDG-1 mice were significantly higher than those in HF group. We thus inferred that MDG-1 may reduce the serum TG content and regulate the oxidation of fatty acids by stimulating the expression of PPARα and its target genes. In addition, Gu et al. [33] found that inhibiting *PPARγ* could prevent HFD-induced hyperlipidemia. HFD caused an overexpression of *PPARγ* in liver tissue with steatosis; thus, the deletion of *PPARγ* could protect against HFD-induced obesity and IR in mice [34,35,36]. In our research, MDG-1 could decrease the expression of *PPARγ* that is involved in glucose and lipid metabolism, implying MDG-1 may be a potential regulator of *PPARγ*.

There are 3 core ways that the body carefully regulates to maintain the levels of cholesterol: through synthesis, catabolism, and excretion. A permanent high-cholesterol diet keeps the synthesis of cholesterol at a stagnant level, but it also strongly activates the catabolism and excretion of cholesterol to achieve a homeostatic balance [37]. *LXRα* is a major hepatic regulator of cholesterol absorption, transport, efflux, and excretion, and mediates cholesterol homeostasis of the body. *LXRα* is also a *PPARα* and *PPARγ* target gene [38,39,40]. Besides, the catabolism of cholesterol involves its breakdown into bile acids and 7α-hydroxylase (*CYP7A1*), and is the rate-limiting step in this biochemical pathway [41]. In the research, when the intake of cholesterol in the body increases, MDG-1 could significantly increase the activity of LXRα and then increase the transcriptional activity of *CYP7A1*, thereby accelerating the transformation of cholesterol to bile acids and reducing the body cholesterol levels [42]. The results were generally consistent with our previous study that MDG-1 could regulate the synthesis, secretion, and reabsorption of bile acids.

It was also observed that under the intervention of MDG-1, the expression of *SREBP-1c* was markedly decreased as compared to the HFD group. The sterol regulatory element-binding proteins (SREBPs) regulate lipid homeostasis and are present in three isoforms: *SREBP-1a*, *SREBP-1c*, and *SREBP-2*, with the second form being the predominant one [43]. Researches show that the transcriptional activity and levels of *SREBP-1c* are strongly associated with hepatic TG accumulation, insulin resistance, and metabolic dysfunction [44,45,46]. This study found that MDG-1 could significantly improve insulin resistance; we suspect that this improvement could be accounted for through the down-regulation of SREBP expression. At the same time, LXRα was activated, which reminds us that MDG-1 not only reduced the cholesterol content of the body but also decreased the generation of fat.

In conclusion, MDG-1 regulates partial core pathways closely related to lipid metabolism. Furthermore, MDG-1 interacts with peroxisome proliferator-activated receptors (PPAR) α and γ directly, activating *PPARα* whilst inhibiting *PPARγ*, thus promoting the metabolism of cholesterol. Besides, MDG-1 could up-regulate the expression of *LXRα* and *CYP7A1* affiliating to PPARs, and inhibit the levels of *SREBP-1c*, thereby promoting cholesterol uptake and excretion. Hence, MDG-1, as a regulator of *PPARα* and *PPARγ*, possesses significant importance in a myriad of biochemical processes such as the regulation of blood lipid metabolism and hyperlipidemia.

## 4. Materials and Methods

### 4.1. Chemical and Diet

Chemical MDG-1 was extracted from the radix of *O. japonicas* and purified according to our previously reported method [18]. High-fat diets (60% of calories derived from fat) and low-fat diets (10% of calories derived from fat) were purchased from Research Diets Inc. (D12492, D12450B, New Brunswick, NJ, USA).

### 4.2. Animals and Treatment

Eight-week-old C57BL/6J male mice (the animal experiment was approved by the Animal Ethical Experimentation Committee of Shanghai University of Traditional Chinese Medicine and an ethic approval number SZY201511001 was got in November 2015.) were purchased from the Beijing Vital River Laboratory Animal Technology Co., Ltd. (Beijing, China) and kept under a constant controlled temperature (22 ± 3 °C) and on a 12 h light/dark cycle, with free access to water and food during the experiment. The mice were randomly divided into three groups on the basis of body weight for preventive treatment: Con (low-fat diet, *n* = 8); HFD (high-fat diet, *n* = 8); and HFD + MDG-1 (5‰ MDG-1 was mixed into the high-fat diet, *n* = 8). The whole experiment lasted for 12 weeks. During the whole experiment, food intake and body weight were recorded and calculated three times a week. The animals’ protocol was carried out according to the guidelines of the Animal Ethical Experimentation Committee of Shanghai University of Traditional Chinese Medicine, and all procedures were in compliance with the National Institutes of Health Guide for Care and Use of Laboratory Animals (Publication No. 85-23, revised 1985).

### 4.3. Serum Chemistry Analysis

After a 12 h fast, the mice were given each an intraperitoneal injection of 20% urethane solution and blood samples were taken. The serum samples were collected and isolated from the blood samples after standing for 2 h at 4 °C and centrifuging at 3000 rpm for 10 min at 4 °C. Using a Hitachi 7020 Automatic Analyzer (Hitachi Ltd., Tokyo, Japan), serum triglyceride (TG), total cholesterol (TC), HDL cholesterol (HDL-c), and LDL cholesterol (LDL-c) were determined by using 100 μL of blood serum.

### 4.4. Liver and Fecal Lipid Contents Analysis

The liver tissues were weighed and homogenized in a 0.5 mL tissue lysis buffer (20 mM Tris-HCl pH 7.5, 150 mM NaCl, 1% Triton) and extracted with an equal volume of chloroform. The chloroform layers were dried and dissolved in isopropyl alcohol to measure lipid levels as described [47].The fecal samples were weighted and milled with PBS solution. The next steps concluded the extraction and measures were same as the above description and the previous reports [47,48].

### 4.5. Hematoxylin and Eosin (H&E) Staining and Scanning Electron Microscopy

Hematoxylin and eosin (H&E) staining: for H&E staining, the tissue was fixed in 10% formaldehyde, embedded in OCT compound and cut into a 10 mm section according to a standard protocol. The procedures of H&E staining mainly include the following steps: fixing, refrigeration and embedding, modest thawing, rapid sectioning, section flatting, and staining. At last, the sections stained with hematoxylin and eosin were examined under a light microscope.

Scanning Electron Microscopy: scanning electron microscopy was used to examine the structure of fat tissue. According to the related protocols [49], the adipose tissue was fixed in 1% osmium tetraoxide. The images were taken using a Philips XL-30 scanning electron microscope.

### 4.6. Glucose Tolerance and Insulin Tolerance Tests

Glucose tolerance tests: at end of the whole experiment, after the mice were fasted for 12 h, the fasting glucose levels were determined from the tail vein (0 min) before the injection of glucose. Additional blood samples were collected at regular intervals (15, 30, 60, 90, and 120 min) for glucose measurements following the injection of glucose (1 g/kg body weight).

Insulin tolerance tests: without the fasting of mice, mice were injected 0.3405 uL/mL insulin (0.75 U/kg body weight) by intraperitoneal, then measured at blood glucose levels of 0, 15, 30, 60, 90, and 120 min intervals.

### 4.7. Gene Array Experiment

#### 4.7.1. RNA Preparation and cDNA Generation

Total RNA was extracted from the liver tissue samples using the TRizol Reagent and NucleoSpin^®^ RNA Clean-up Kits and purified using the MessageAmp™ Premier RNA Amplification Kit (Thermo Fisher Scientific). Then, cDNA was synthesized by reverse transcription according to the instructions of one Poly-A RNA Control Kit (besides, cDNA was labeled with fluorescence and purified by purification column).

#### 4.7.2. Gene Array Experiment and Gene Array Data Analysis

The gene chip was constructed according to the manufacturer’s instructions and previous studies [50,51]. The gene expression was detected by the Affymetrix scanner (GeneChip^®^ Scanner 3000, Thermo Fisher Scientific). Based on the RMA algorithm, the data could be analyzed by differential expression gene analysis and another series of follow-up analyses.

### 4.8. Quantitative Real-Time PCR Analysis of Animal Tissues

The protocol of RT-PCR followed the published methods [19,48]. Briefly, the liver and white fat tissues (around 100 mg) were homogenized (65 Hz, 2 min) in 1 mL RNAiso Plus. Then, total RNA was extracted according to the previous description. The cDNA was synthesized using a cDNA synthesis kit (Applied Biosystems, Grand Island, NY, USA), and gene expression levels were measured by quantitative real-time RT-PCR using the ABI Step one Plus Real-Time PCR system (Applied Biosystems). The primers used in the experiments are shown in Table 1.

### 4.9. Stastical Analysis

All data were presented as means ± SD and analyzed using SPSS 21.0 for Windows. Statistical analysis was performed using one-way analysis of wariance (ANOVA). In the study, *p* < 0.05 showed significant difference when *p* > 0.05 represented not significant. All data were illustrated by GraphPad software (Inc., San Diego, CA, USA).

## 5. Conclusions

In summary, we found that MDG-1could prevent the development of obesity and ameliorates dyslipidemia in HFD-induced obese mice. These effects appear to be mediated through the inhibition of *PPARγ* and activation of *PPARα* as well as regulation of their target genes. Thus, our results suggest that MDG-1 may have remarkable function of adjusting the serum lipid.

## Figures and Tables

**Table 1:** Primer sequences of Real-time PCR.

Gene	Forward Primer	Reverse Primer
*β-Actin*	TGTCCACCTTCCAGCAGATGT	AGCTCAGTAACAGTCCGCCTAGA
*PPARα*	AGGCTGTAAGGGCTTCTTTCG	GGCATTTGTTCCGGTTCTTC
*PPARβ*	AGTGACCTGGCGCTCTTCAT	CGCAGAATGGTGTCCTGGAT
*PPARγ*	CGCTGATGCACTGCCTATGA	AGAGGTCCACAGAGCTGATTCC
*LXRα*	GAGTGTCGACTTCGCAAATGC	CCTCTTCTTGCCGCTTCAGT
*LXRβ*	CAGGCTTGCAGGTGGAATTC	ATGGCGATAAGCAAGGCATACT
*Cyp7a1*	GTGGTAGTGAGCTGTTGCATATGG	CACAGCCCAGGTATGGAATCA
*CYP8B1*	GGACAGCCTATCCTTGGTGA	GACGGAACTTCCTGAACAGC
*SREBP-1c*	GGCTATTCCGTGAACATCTCCTA	ATCCAAGGGCATCTGAGAACTC
*FAS*	CTGAGATCCCAGCACTTCTTGA	GCCTCCGAAGCCAAATGAG
*ACC-1*	GAATCTCCTGGTGACAATGCTTATT	GGTCTTGCTGAGTTGGGTTAGCT
*aP2*	CATGGCCAAGCCCAACAT	CGCCCAGTTTGAAGGAAATC
*ApoE*	GAACCGCTTCTGGGATTACCT	TCAGTGCCGTCAGTTCTTGTG
*CD36*	GCTTGCAACTGTCAGCACAT	GCCTTGCTGTAGCCAAGAAC

## Abbreviations

HFD	High-fat diet
DIO	Diet-induced obese
TC	Total cholesterol
TG	Total triglyceride
HDL-c	High-density lipoprotein cholesterol
LDL-c	Low-density lipoprotein cholesterol
PPARs	Peroxisome proliferator-activated receptors
SCFAs	Short-chain fatty acids
qPCR	Quantitative real-time PCR
H&E staining	Hematoxylin and eosin staining
